# Gender Differences in Eating Habits of Polish Young Adults Aged 20–26

**DOI:** 10.3390/ijerph192215280

**Published:** 2022-11-18

**Authors:** Marian Gil, Mariusz Rudy, Renata Stanisławczyk, Paulina Duma-Kocan, Jagoda Żurek

**Affiliations:** 1 Department of Agricultural Processing and Commodity Science, Institute of Food and Nutrition Technology, College of Natural Sciences, University of Rzeszow, Zelwerowicza 4, 35-601 Rzeszów, Poland; 2 Department of Financial Markets and Public Finance, Institute of Economics and Finance, College of Social Sciences, University of Rzeszow, Cwiklinskiej 2, 35-601 Rzeszów, Poland

**Keywords:** dietary habits, analysis of consumption, number of meals consumed

## Abstract

The aim of the study was to examine the nutritional behaviour of young adults depending on gender. A survey was conducted among 467 young adults using the “Questionnaire for the study of nutritional behaviour and opinions on food and nutrition”. Questions concerned the frequency of consumption of selected groups of food products. The questionnaire was supplemented by questions regarding the number of portions of fruits and vegetables consumed, putting sugar in drinks, putting salt in dishes and the number of glasses of water drunk. Differences in nutritional behaviours were determined using the χ^2^ test, at *p* < 0.05. The dietary choices of women more often than those of men corresponded to the principles of healthy nutrition, related to a greater number of meals consumed during the day, more frequent consumption of fruits and vegetables and the selection of products with lower energy value or preferring healthier methods of culinary processing. Health education programs should prevent the emergence of unfavourable dietary habits such as skipping breakfast or other meals or limiting the consumption of fruits and vegetables and frequently replacing them with high-energy snacks.

## 1. Introduction

Dietary habits are a main aspect of a lifestyle affecting health, morbidity and mortality in various conditions [1]. A healthy diet helps to protect against malnutrition in all its forms, as well as non-communicable diseases such as diabetes, heart diseases, stroke and cancer [2]. The assessment of trends in dietary intake is essential for the early detection of nutritional problems within the entire population [3,4].

Research on dietary habits in children and youth shows many negative habits [5]. Dietary habits shaped during adolescence are often continued in adulthood. The change in the environment that is associated with the transition of students from secondary school to university enforces the need to adapt to new conditions, greater responsibility for dietary choices and practices [6]. Therefore, it is important to shape appropriate dietary habits and dietary behaviour during adolescence in order to prevent diseases related to lifestyle in adulthood [7,8]. Weight gain and poor dietary patterns during studies (20–26 years) may have adverse physiological consequences and lead to late-onset chronic diseases such as overweight and obesity, cardiovascular disease, type 2 diabetes [9] and, in consequence, lead to increased costs of health care [10,11].

The key issues are food consumption patterns and related nutritional risks specific to students [12,13]. Studies of eating habits in young adults have shown a lot of unhealthy eating practices, especially in students living away from home [14,15,16]. According to research conducted at American universities, first-year students significantly reduced the consumption of bread and vegetables, which should be assessed as a negative phenomenon due to the fact that these food groups are an important source of fibre, vitamins and minerals. Conversely, replacing meals with high-calorie snacks contributes to higher fat consumption. The fats in the snacks contain trans fatty acid isomers which can cause cardiovascular disease [17].

The diet of young adults is influenced by their financial situation, products offered by gastronomy, individual tastes and the level of nutritional knowledge. The lifestyle of this group is characterized by irregularity. Work and studies take up a lot of time, which contributes to reducing the number of meals or replacing them with snacks. The eating habits determined by the organoleptic qualities of the food often strengthen the deficiencies in the diet [18,19]. Skipping or irregularly eating meals results in skipping food essentials for health or using snacks more frequently to temporarily satisfy hunger, thus providing large amounts of simple sugars and fats. People skipping at least one main meal a day consume smaller amounts of fruits and vegetables compared to those who do not skip any meals [20,21,22]. The suboptimal health behaviours in young adults may change into long-term behavioural patterns and may remain throughout the entire adult life. That is why preventive programs are needed to counteract unhealthy eating habits among this group to prevent more frequent overweight and obesity in later life. Young adults are an important target for nutritional education interventions. To develop effective strategies to prevent obesity, it is important to gain insight into factors affecting the nutritional behaviour of young adults [17,23]. The Multi-centre National Population Health Examination Survey (WOBASZ study) was carried out in Poland in 2003–2005 on a randomly selected sample of residents from all over Poland aged 20 years or older (14,537 people) and in 2013 and 2014 (WOBASZ II study—6164 people). The results of the WOBASZ II study indicate a very high prevalence of hyperlipidaemia, hypertension, obesity, metabolic syndrome and diabetes in the adult population in Poland, as well as a high level of global risk. There was a significant increase in the incidence of the metabolic syndrome in adult Poles aged 20 to 74 years by 3.3% in women (26.6% vs. 29.9%) and by 8.8% in men (30.7% vs. 39.4%). The following components of metabolic syndrome were higher for men: increased level of triglycerides, increased blood pressure or hypertension and increased fasting blood glucose or diabetes. The following components of metabolic syndrome were higher for women: incidence of weight gain and reduced HDL-C levels [24,25]. There has been an increase in the prevalence of obesity in the entire population, especially in men [26]. Stepaniak et al. [26] pointed to the need to undertake more effective public health campaigns to prevent and treat obesity, especially in the case of men and young adults.

Due to their subsequent influence on shaping attitudes, it is necessary to implement healthy eating behaviours among young adults [18]. Incorrect frequency and amount of dietary components consumed may increase the incidence of diseases, while the presence of other components in the diet contributes to their reduction [27]. Nutritional knowledge is considered a factor contributing to the occurrence of recommended behaviours [19].

In many works, the authors point to differences depending on gender in observing the principles of a healthy lifestyle, including eating behaviours [28,29,30,31].

The aim of the study was to examine the nutritional behaviour of young adults depending on gender.

## 2. Materials and Methods

### 2.1. Research Project and Sample

The analysis was based on the data from the questionnaire: “Questionnaire for the study of dietary behaviour and opinions on food and nutrition” KomPAN developed by the Team for Behavioural Conditions of Nutrition of the Committee for Human Nutrition Science of the Polish Academy of Sciences [32].

The examination of internal consistency of the questionnaire was carried out by testing the repeatability (test–retest) in 954 people aged 15–65 (440 men, 514 women), from 5 cities/regions of Poland. Two versions of the questionnaire were analysed: administered by a researcher interviewer (in 299 healthy people) and a self-report (517 healthy people, 138 sick people). The kappa coefficient for the questionnaire in the version administered by the interviewer-researcher in healthy people was greater than 0.60 for all 33 product groups and ranged from 0.62 (cured meat products, sausages, frankfurters) to 0.84 (energy drinks), for eating habits it ranged from 0.71 to 1.00. In healthy subjects, the internal consistency of the KomPAN questionnaire was moderate to very good for individual product groups and both dietary quality indicators and dietary habits, lifestyle characteristics, nutritional knowledge indicators and physical activity, with better consistency shown for the version administered by the interviewer rather than the self-reversing type [33].

In total, 59.9% of the surveyed women lived in rural areas (48.4% of men), 18.0% in towns with less than 20,000 inhabitants (17.9% men), 13.4% in cities with 20–100 thousand inhabitants (14.7% of men) and 8.6% in cities with more than 100,000 inhabitants (18.9% men). The survey was voluntary, anonymous and the respondents were instructed about the principles of filling out the questionnaire. The percentage of correctly completed questionnaires was 93.4%. In total, 33 incomplete surveys were rejected. The research covered 467 young adults. Respondents were aged 20–26. The respondents declared that the energy value of their daily diet ranged from 2000 kcal to 2500 kcal. Moreover, the respondents declared that the share of energy from carbohydrates, fats and proteins was as follows: 50–70%—carbohydrates; 20–35%—fats; 10–15%—proteins.

### 2.2. Measures

#### Nutrition Rating

Respondents answered questions about the number of meals during the day, the regularity of their consumption and eating between meals. The questions concerned the frequency of consumption of the following groups of food products: wholemeal bread, milk, milk fermented beverages, cottage cheese, hard cheese, meat products and meat dishes, fish products and fish dishes, legume seed dishes, potatoes, fruits, vegetables, fruit and vegetable juices, fast food and fried dishes. The frequency was assessed according to the following scale: never, 1–3 times a month, once a week, several times a week, once a day and several times a day. The questionnaire was supplemented by questions regarding the number of portions of fruits and vegetables consumed, putting sugar in drinks, putting salt in dishes and the number of glasses of water drunk.

In order to standardize the interpretation of the results, there were used the daily frequency indicators expressed as times/day. In order to comprehensively assess the quality of the diet, the indicators were calculated, one of which includes foods with a potentially beneficial effect on health, and the other includes foods detrimental to health: “Pro-Healthy Diet Index” (pHDI-10, Pro-Healthy-Diet-Index-10), “Non-Healthy Diet Index” (nHDI-14, Non-Healthy-Diet-Index-14) [34].

In order to standardize the scope of both indexes and to facilitate their interpretation, the total frequency of consumption (times/day) and its expression on a scale from 0 to 100 points were recalculated.

“Pro-Healthy Diet Index” (pHDI-10, in points) = (100/20) × the sum of the frequency of consumption of 10 food groups (times/day).

“Non-Healthy Diet Index” (nHDI-14, in points) = (100/28) × the sum of the frequency of consumption of 14 food groups (times/day).

The pro-healthy diet index (pHDI-10, Pro-healthy-Diet-Index-10) was calculated by summing the frequency of consumption (multiples/day) of the following food groups: (1) wholemeal bread, (2) buckwheat, oatmeal, whole wheat pasta or other coarse groats, (3) milk (including flavoured milk, cocoa, coffee with milk), (4) fermented milk drinks, e.g., yoghurts, kefirs (natural or flavoured), (5) cottage cheese (including homogenized cheese, cottage cheese desserts), (6) dishes from the so-called white meat, e.g., chicken, turkey, rabbit, (7) fish, (8) legume seeds dishes, e.g., beans, peas, soya beans, lentils, (9) fruit, (10) vegetables.

The non-healthy diet index (nHDI-14, Non-Healthy-Diet-Index-14) was calculated by summing up the frequency of consumption (multiple/day) of the following food groups: (1) light bread, e.g., wheat, rye, mixed wheat-rye, toasted bread, rolls, croissants, (2) white rice, plain pasta or small groats, e.g., semolina, couscous, (3) fast food, e.g., French fries, hamburgers, pizza, hot dogs, baguettes casseroles, (4) meat or flour fried dishes (5) butter as an addition to bread or dishes, for frying, baking, etc., (6) lard as an addition to bread or dishes, for frying, baking, etc., (7) yellow cheese (including processed cheese, blue cheese), (8) cured meat products, sausages or frankfurters, (9) dishes from the so-called red meat, e.g., pork, beef, veal, mutton, lamb, venison, (10) sweets, e.g., candies, cookies, cakes, chocolate bars, muesli bars, other confectionery products, (11) canned meat, (12) sweetened carbonated or non-carbonated drinks such as Coca-Cola, Pepsi, Sprite, Fanta, orangeade, lemonade, (13) energy drinks, e.g., 2 KC, Black Horse, Red Bull, Burn, Shot or others, (14) alcoholic drinks.

The interpretation of indexes is intuitive—the higher the index value, the greater the intensity of the features favourable or unfavourable to health. The idea of interpreting is the same for indexes expressed as total times/day or in points. The proposed interpretation of the indexes is presented in Table 1.

The calculated indexes of pro-healthy diet were significantly higher in the group of women, while the index of non-healthy diet had higher values in the group of men. The intensity of the nutritional characteristics in relation to both the pro-healthy diet and the non-healthy diet should be described as low.

#### 2.3. Data Analysis

A descriptive analysis was carried out regarding the dietary habits of the analysed population. Differences in nutritional behaviour were determined using the χ^2^ test, at *p* < 0.05. Statistical analyses were performed using the statistical package STATISTICA 13.5PL (StatSoft Polska Sp. z o.o., Krakow, Poland).

## 3. Results

The study was conducted on respondents aged 19–25 (Table 2). The average age of the surveyed respondents exceeded 22 years. Weight and height were self-reported by study participants. The height and weight of men and women were different in a statistically significant way. The average BMI (Body Mass Index) values of the surveyed women were significantly lower than the BMI of men. BMI values in the study population were within the normal proportions between height and weight.

Half of the women consumed four meals a day, and every third woman had three meals (Table 3). Consuming two meals a day was declared by a small number of women. The distribution of the number of meals consumed by men looked a bit different. It was found that a lower percentage of men than of women consumed four and five or more meals, while a higher percentage of men declared eating three and two meals a day.

More than half of the women reported that they do not eat meals regularly, while in the case of men, two out of three respondents indicated the same. Regular consumption of some meals was declared by a greater number of women (37.4%) than men (31.6%). Eating meals at regular hours was declared by every tenth female and one in thirty men.

**Figure 1:**
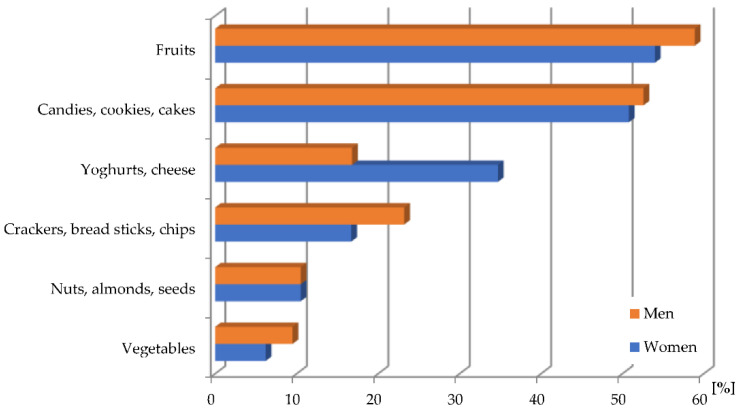
Most frequently chosen snacks by male and female [%].

Significant irregularity of consumption is connected with the scale of snacking between meals. In the analysed population, this phenomenon is widespread (87.9% of women, 88.4% of men). Consumption of food between meals a few times a week was declared by a larger percentage of women (30.1%) than men (25.3%). One in four respondents in the analysed population snacks once a day, the same proportion of men report to snacking a few times a day. Among women, every fifth respondent declared snacking several times a day. Both women (54.0%) and men (58.9%) indicated the foods that they most frequently chose for snacks were fruits (Figure 1). The second most frequently consumed snack category was candies, cookies and cakes. Only every third woman and every sixth man declared the consumption of yoghurt or cheese. Crackers, breadsticks or crisps between meals were consumed more often by men than women. Vegetables as well as nuts, almonds or seeds are consumed by a small group of respondents, and these are groups of valuable produce and could enrich the diet with valuable and often scarce nutrients.

Table 4 shows the frequency of consumption of selected food groups depending on the gender of young adults. The consumption of fast food was small. The vast majority of women declared consumption of such products 1–3 times a month (77.2%) and once a week (14.2%). In the group of men, fast-food products were consumed 1–3 times a month by 46.3% and once a week by 26.3% of the respondents.

Fried products are sporadically consumed by a large number of women, 1–3 times a month by 12.1% and once a week by 33.3% of the respondents. Consumption of these products several times a week was declared by 51.1% of women and 45.3% of men.

From the point of view of healthy nutrition, it is important to consume wholemeal bread [35]. This type of bread was consumed more often by women. It was consumed once a day or more often by 20.9% and several times a week by 28.8% of women. Wholemeal bread was consumed at least once per day by 13.7% of men and 13.7% of women. Consumption of milk 3 times a month or less was declared by 29.3% of women and 34.7% of men. Milk was consumed several times a week by 31.5% of women and 24.2% of men. Among women, milk was consumed once a day or more often by 21.5% of the group, and only 12.6% of men consumed milk with such frequency. More frequent consumption of milk fermented beverages was declared by women. Consumption of these products several times a week was declared by 46.8% of women, and once a day by 12.4%, whereas among men it was 16.8% and 14.7%, respectively. Cottage cheese was consumed several times a week by 43.8% of women and 31.6% of men, while hard cheese was consumed several times a week by more men (50.5%) than women (39.0%). Consumption of meat products and meat dishes once a week was declared by 19.9% of women and 6.3% of men, several times a week by 62.4% of women and 56.8% of men. A larger percentage of men (14.7%; 11.0% of women) declared consumption of this product group on a daily basis, and 20.0% of men (1.3% of women) consumed meat products and meat dishes several times a day.

Fish products and fish dishes were rarely consumed by respondents. Consumption of these products several times a week was declared by 8.3% of women and 11.6% of men. Once a week, fish dishes were consumed by 30.6% of women and 34.7% of men. Sporadic consumption of these products 1–3 times a month was declared by 56.7% of women and 41.1% of men.

Dishes from legume seeds such as beans or peas were consumed 1–3 times a month by the vast majority of young adults (69.4% of women; 73.7% of men), and a group of respondents did not eat them at all (13.2% of women; 15.8% of men).

More women than men declared consuming potatoes once a week (25.8% vs. 15.8% for men), 1–3 times a month (15.6% vs. 7.4% for men) and never (7.3% vs. 3.2% for men). In contrast, in the group of men the following answers predominated: several times a week (57.9% vs. 48.7% for women), once a day (11.6% vs. 2.7% for women) and even more frequently (4.2% vs. 0.0% for women).

For fruit consumption, the largest group of women (44.4%) consumed fruits several times per week. For fruit consumption, the largest group of women (44.4%) consumed fruits several times per week. Daily consumption of fruits was declared by 22.3% of women, and fruits were consumed several times a day by 19.1% of women. The proportion of men consuming fruits several times a week was lower than in women and it was 38.9%, more men than women consumed fruits once a day (30.5%), and only 5.3% consumed them several times a day. Among men, 25.2% consumed fruits only once a week or less, in women it was 14.2% fruits were consumed several times a day.

Consumption of vegetables several times a week was declared by half of the respondents. Consumption of vegetables once a day was indicated by a higher percentage of women (18.5%) than men (13.7%), and consumption of vegetables several times a day was declared by twice as many women (21.2%) as men (9.5%).

The recommended number of five portions of fruits and vegetables or more per day was consumed by only a small group of women (4.0% vs. 0.0% for men), and four portions a day by a slightly higher percentage (5.9% vs. 2.1% for men) (Table 5). Those who consumed three portions of fruits and vegetables constituted 18.5% of women and 9.5% of men. The largest group of women consumed two portions of fruits and vegetables a day—45.4%, among men it was 36.8%. Consumption of only one portion a day was declared by 46.3% of men.

In consumption of fruit and vegetable juices among respondents, no significant differences were found in terms of gender: the most numerous groups of women (30.5%) and men (30.6%) declared consumption of juices several times a week. As in the case of juices, the amount of water drunk did not depend on gender. Six glasses of water and more were drunk by 20.3% of respondents, and 23.8% indicated drinking four glasses of water a day.

Sweets were consumed once a week or less by 32.7% of women, and in the group of men it was 46.3%. The majority of women (50.5%; 27.4% of men) consumed sweets several times a week, but the percentage of persons consuming sweets each day was lower (11.3%) than in men (17.9%). Men also put more sugar in hot drinks such as coffee or tea. The use of two or more teaspoonfuls of sugar was indicated by 71.6% of men, and the same was indicated by 22.8% of women (Table 6). Among the surveyed woman, 46.8% did not put sugar in beverages, and among men, only 20.0% of respondents did not put sugar in beverages.

Regularly putting salt in ready meals is practiced more often by men (16.8%) than women (6.2%). Occasional addition of salt was similar among respondents and was practiced by 41.1% of men and 39.5% of women, whereas a larger group of women did not add salt to ready meals (54.3% vs. 42.1% for men).

The value of the pro-healthy diet index (pHDI-10) in the group of women was 4.63 and was significantly higher than in the group of men (3.57). The value of the non-healthy diet index (nHDI-14) was significantly lower in the group of women (5.05) than in the group of men (6.39).

## 4. Discussion

In order to prevent nutritional problems, it is important to identify and foresee changes in nutritional behaviour in particular population groups. The need to monitor dietary habits among young people has intensified in recent years due to the growing epidemic of overweight around the world [4]. In the study by Błaszczyk-Bębenek et al., abdominal obesity was found in 10.7–21.7% of the Polish adolescents surveyed. There was an association between skipping breakfast and the incidence of central obesity [36].

Commencing studies and living away from home is connected with a change in living conditions that can lead to reorganization of nutritional behaviour. Many students are for the first time in their life completely responsible for buying food and planning or preparing meals [5,6,37,38]. This is due to the decrease in involvement of children and youth in preparation of family meals, which results in the lack of cooking skills among young adults [31].

A negative phenomenon more often observed is skipping one or more main meals (breakfast, lunch or dinner) [39,40,41]. In the present study, three meals and less were consumed by 35.5% of women and 46.3% of men. The review of the studies by Pendergast et al. [37] shows that the most often skipped meal for the 18–30 age group was breakfast, and the most common reason was lack of time. Skipping meals was dependent on gender—men more often skipped breakfast, and women lunch or dinner. The estimated frequency of skipping meals in the young adult population ranges from 24 to 87% and is higher compared to other age groups [41]. Kowalska and Tarnowska [31] showed that women more often declared healthy eating. Most students ate 3–4 meals a day, and regular breakfasts were declared among 42 percent of respondents. The consumption of sweet snacks between meals was confirmed by half of the respondents. Students of food sciences more often than others thought that they ate healthily, although students of non-food lines most often declared having five or more meals a day. In the studies of Szponar and Krzyszycha [42], it was stated that among students of the Medical University of Lublin, every fifth student would eat sweets several times a day, more than half of students would eat sweets several times a week and 17% once a day. For comparison, the intake of sweets among students of the dietetics of the University of Natural Sciences and Humanities in Siedlce was clearly lower: 6% of women students ate every day, and 18% ate sweets a few times a week [43]. They also found in this group a higher intake of 3–4 meals a day (67% of women students) and 30% had 5 meals. Having 4 and more meals was declared by 80% of students of the Faculty of Health Sciences in Białystok [44]. According to Skibniewska, having three and four meals during the day was declared by 41% and 38% of Polish students and 46% and 39% of Belgian students [45]. The Orkusz study [46], comparing the number of eaten meals between women and men, shows more frequent consumption of four meals (62.9% women, 63.2% men) and, respectively, a lower consumption of three meals (23.4%, 22.8%).

Skipping breakfast in childhood and adulthood [38,47] results in a higher level of insulin on empty stomach, higher cholesterol level, BMI and a larger waist circumference compared to people eating breakfast [39,48,49]. Therefore, eating breakfast is considered to be health promoting behaviour [50] bringing various health benefits [50,51].

Orkusz’s research [46] showed that 12.7% of surveyed students (12.1% of women and 14.0% of men) declared irregular or missing first breakfast. However, research conducted among students of the Medical Academy in Wroclaw [51] and the Medical University of Lublin [42] showed that a larger percentage of men students (25.7% and 50.8%, respectively) than women students (respectively 16.8% and 39.9%) do not have the first breakfast or have it irregularly. Likus et al. [52] showed that in the group of students of the Silesian Medical University in Katowice, 25% of respondents did not have first breakfast.

The literature on nutritional behaviour indicates significant differences in food choices between men and women. Women consume more fruits and vegetables, have a higher intake of dietary fibre and a lower intake of fat. In addition, the motivation to control weight is more visible in women, which increases their involvement in healthy dietary habits [8,29,30,37,38]. The cause of differences between the genders can be generally higher health awareness [9,28], better knowledge about nutrition [53] and better knowledge of what constitutes a “healthy diet” [54] among women.

Szczuko et al. [6] indicate a higher effectiveness of education in the field of healthy nutrition for women than for men. Jeżewska-Zychowicz and Zięba [9] investigated the influence of factors determining fruit and vegetable consumption among students of the first and last years of nutrition science and confirmed the significant influence of nutritional knowledge on the consumption of certain vegetables and fruits among students. Szczęsna et al. [10] assessed the influence of nutritional knowledge on the preferences and nutritional behaviours of students of various faculties at the Agricultural University. They showed that the students of the food technology department more often consumed fruits and vegetables in comparison with the students of other faculties. In the case of fast food, the situation was the opposite—students of other departments more often consumed French fries, casseroles and hot dogs than students of food technology.

Popławska et al. [55] studied dietary habits of students of the first and last year of physical education academy. A comparison of students in terms of level of education showed that last year students more often consumed poultry, fruit and vegetables, while first-year students more often consumed white bread, butter, cold meats, cheese and sweets. Women with a high level of physical activity more often consumed healthy food, whereas men showed such a tendency only in the case of dairy products. The majority of students showed sufficient knowledge about food and nutrition, and it was observed that their university studies increased the level of knowledge in this respect.

This research confirms the differences in consumption of fruits and vegetables depending on gender. Significant differences were found in the number of portions of fruits and vegetables during the day: four and more portions were consumed by 30.6% of women and only 7.4% of men. Additionally, in the research by El Ansari et al. [5], Polish students reported a low consumption of vegetables (15.2%) and fruits (35.0%).

The level of vegetable consumption in the EU ranges from 20% to 54.0% of daily recommendations [56]. In fact, consumption of fruits and vegetables is higher in southern Europe [57]. In Denmark, the average consumption of fruits and vegetables per day is 53.0% of the 600g recommended daily intake. Therefore, the existing consumption of fruits and vegetables, lower than recommended, requires effective interventions [58].

According to studies conducted at American universities [59,60,61], students do not consume the recommended amount of fruits and vegetables and they ate more and more foods with high content of fat. In Europe, the same pattern of weight gain in students appears [15]. In Greece, students living away from family home made some positive changes (e.g., a decrease in the content of full-fat dairy products, white bread and margarine), but, at the same time, they reduced consumption of fresh fruits, boiled and raw vegetables, fatty fish, and increased consumption of sugar and fast food [5,62,63]. Similarly, Bulgarian students reported frequent consumption of sweets (52.8%), cakes (60.9%) and snacks (77.1%) (for example, crisps and fast food). In Poland, Denmark and Germany, men consumed snacks more often than women. Students living in their parents’ home consumed more fruits, vegetables and meat than those who lived outside family home in all analysed countries [5].

Studies show that people experiencing stress reported that they overate foods they would normally avoid and that they eat these foodstuffs to feel better [1,64]. The connection between unhealthy dietary habits and higher levels of stress was also found by other researchers who showed that under stress people more often consumed sweets, chocolate, cake and biscuits [65,66], high-fat food and fast-food snacks [67].

Many studies [1,18,19,54] indicate that women more frequently consume boiled vegetables, salads/raw vegetables, fresh fruits and cottage cheese/cream cheese/yoghurts, while consumption of red meat, poultry, sausages, fish and hard/soft cheese was more frequent among male students. In addition, men more often than women consumed fast food and such dishes as pasta/rice and fried potatoes/crisps. Differences between the genders were also observed in the frequency of chocolate consumption, and women consumed chocolate more often than men.

In the work of Gacek et al. [68], the mean values of the pHDI10 index determined for Polish students were 4.92 and for Spanish students 4.35. Non-healthy diet index defined in the work of Gacek et al. [68] was 4.45 for Polish students and 3.74 for students from Spain.

Therefore, it is important to counteract unhealthy dietary habits in university students and promote appropriate dietary habits and nutritional behaviour to prevent diseases related to adult lifestyle. In order to develop effective strategies to prevent obesity, it is important to learn about determinants of nutritional behaviour in university students [7,17].

A limitation of this work is the exclusion of questions relating to the quality of diet, e.g., regarding preferred products consumed in the course of individual dishes, methods of culinary processing. However, the work focuses mainly on the aspect of frequency of consumption of products. In theory, students’ dietary habits may be conditioned by local dietary habits.

## 5. Conclusions

Gender was a factor differentiating the dietary habits of young adults. The nutritional choices of women more often than men corresponded to the principles of healthy nutrition. Women had more meals during the day, and vegetables were consumed more often. Women chose products of lower energy value more often and preferred healthy cooking methods. Health education programs should prevent the emergence of unfavourable eating habits such as skipping breakfast or other meals or limiting the consumption of fruits and vegetables and replacing them with often high-energy snacks.

## Figures and Tables

**Table 1:** Proposed interpretation of the “Pro-Healthy Diet Index” (pHDI-10) and the “Non-Healthy Diet Index” nHDI-14) for the KomPAN Questionnaire [34].

The Intensity of Nutritional Characteristics	Scope (Times/Day)		Scope (in Points)	
	“Pro-Healthy Diet Index” pHDI-10	“Non-Healthy Diet Index” nHDI-14	“Pro-Healthy Diet Index” pHDI-10	“Non-Healthy Diet Index” nHDI-14
Low	0–6.66	0–9.33	0–33	0–33
Moderate	6.67–13.33	9.34–18.66	34–66	34–66
High	13.34–20.00	18.67–28.00	67–100	67–100

**Table 2:** Age, weight and height of respondents.

Specification	Total *n* = 467	Women *n* = 372	Men *n* = 95
Age (years)	22.4 ± 1.32	22.3 ± 1.40	22.5 ± 0.90
Height (cm)	169.1 ± 7.69	166.7 ± 6.11 ^a^	178.4 ± 5.94 ^b^
Body weight (kg)	61.7 ± 11.82	58.0 ± 8.47 ^a^	76.2 ± 11.88 ^b^
BMI (kg/m^2^)	21.5 ± 3.18	20.9 ± 2.82 ^a^	23.9 ± 3.35 ^b^

^a,b^—means marked with different letters differ significantly. BMI—Body Mass Index.

**Table 3:** Gender differences in the number, regularity of meals consumed and snacking of Polish young adults.

Questions	Levels	Total (%)	Women (%)	Men (%)	*p* Value
How many meals do you eat during the day?	1 meal	0 (0.0)	0 (0.0)	0 (0.0)	0.033
	2 meals	12 (2.6)	6 (1.6)	6 (6.3)	
	3 meals	164 (35.1)	126 (33.9)	38 (40.0)	
	4 meals	221 (47.3)	182 (48.9)	39 (41.1)	
	5 meals and more	70 (15.0)	58 (15.6)	12 (12.6)	
Do you eat meals at fixed times of the day?	No	258 (55.2)	196 (52.7)	62 (65.3)	0.031
	Yes. but only some	169 (36.2)	139 (37.4)	30 (31.6)	
	Yes. all	40 (8.6)	37 (9.9)	3 (3.2)	
Do you eat between meals?	No	56 (12.0)	45 (12.1)	11 (11.6)	ns
	Yes	411 (88.0)	327 (87.9)	84 (88.4)	

Differences in behaviour based on the gender of respondents were determined using the χ^2^ test. Statistically significant differences were established at *p* < 0.05.

**Table 4:** Frequency of consumption of selected groups of food products.

Questions	Levels	Total (%)	Women (%)	Men (%)	*p* Value
How often do you eat food between meals?	Never	56 (12.0)	45 (12.1)	11 (11.6)	ns
	1–3 times a month	30 (6.4)	27 (7.3)	3 (3.2)	
	Once a week	31 (6.6)	23 (6.2)	8 (8.4)	
	A few times a week	136 (29.1)	112 (30.1)	24 (25.3)	
	Once a day	123 (26.3)	101 (27.2)	22 (23.2)	
	Several times during the day	91 (19.5)	64 (17.2)	27 (28.4)	
How often do you eat wholemeal bread?	Never	42 (9.0)	29 (7.8)	13 (13.7)	0.0001
	1–3 times a month	155 (33.2)	121 (32.5)	34 (35.8)	
	Once a week	59 (12.6)	37 (9.9)	22 (23.2)	
	A few times a week	120 (25.7)	107 (28.8)	13 (13.7)	
	Once a day	64 (13.7)	53 (14.2)	11 (11.6)	
	Several times during the day	27 (5.8)	25 (6.7)	2 (2.1)	
How often do you consume milk (including flavoured milk)?	Never	52 (11.1)	38 (10.2)	14 (14.7)	0.039
	1–3 times a month	90 (19.3)	71 (19.1)	19 (20.0)	
	Once a week	93 (19.9)	66 (17.7)	27 (28.4)	
	A few times a week	140 (30.0)	117 (31.5)	23 (24.2)	
	Once a day	79 (16.9)	67 (18.0)	12 (12.6)	
	Several times during the day	13 (2.8)	13 (3.5)	0 (0.0)	
How often do you consume fermented milk drinks, e.g., yogurts, kefirs?	Never	7 (1.5)	3 (0.8)	4 (4.2)	0.0001
	1–3 times a month	76 (16.3)	49 (13.2)	27 (28.4)	
	Once a week	122 (26.1)	93 (25.0)	29 (30.5)	
	A few times a week	190 (40.7)	174 (46.8)	16 (16.8)	
	Once a day	60 (12.8)	46 (12.4)	14 (14.7)	
	Several times during the day	12 (2.6)	7 (1.9)	5 (5.3)	
How often do you eat cottage cheese (including homogenized cheese)?	Never	26 (5.6)	23 (6.2)	3 (3.2)	0.001
	1–3 times a month	73 (15.6)	53 (14.2)	20 (21.1)	
	Once a week	125 (26.8)	87 (23.4)	38 (40.0)	
	A few times a week	193 (41.3)	163 (43.8)	30 (31.6)	
	Once a day	33 (7.1)	29 (7.8)	4 (4.2)	
	Several times during the day	17 (3.6)	17 (4.6)	0 (0.0)	
How often do you eat cheese (including processed cheese)?	Never	21 (4.5)	21 (5.6)	0 (0.0)	0.0001
	1–3 times a month	78 (16.7)	70 (18.8)	8 (8.4)	
	Once a week	117 (25.1)	83 (22.3)	34 (35.8)	
	A few times a week	193 (41.3)	145 (39.0)	48 (50.5)	
	Once a day	40 (8.6)	35 (9.4)	5 (5.3)	
	Several times during the day	18 (3.9)	18 (4.8)	0 (0.0)	
How often do you consume preserves and meat dishes?	Never	4 (0.9)	4 (1.1)	0 (0.0)	0.0001
	1–3 times a month	18 (3.9)	16 (4.3)	2 (2.1)	
	Once a week	80 (17.1)	74 (19.9)	6 (6.3)	
	A few times a week	286 (61.2)	232 (62.4)	54 (56.8)	
	Once a day	55 (11.8)	41 (11.0)	14 (14.7)	
	Several times during the day	24 (5.1)	5 (1.3)	19 (20.0)	
How often do you consume preserves and fish dishes?	Never	28 (6.0)	16 (4.3)	12 (12.6)	0.004
	1–3 times a month	250 (53.5)	211 (56.7)	39 (41.1)	
	Once a week	147 (31.5)	114 (30.6)	33 (34.7)	
	A few times a week	42 (9.0)	31 (8.3)	11 (11.6)	
	Once a day	0 (0.0)	0 (0.0)	0 (0.0)	
	Several times during the day	0 (0.0)	0 (0.0)	0 (0.0)	
How often do you eat foods from legume seeds, such as beans and peas?	Never	64 (13.7)	49 (13.2)	15 (15.8)	ns
	1–3 times a month	328 (70.2)	258 (69.4)	70 (73.7)	
	Once a week	47 (10.1)	43 (11.6)	4 (4.2)	
	A few times a week	24 (5.1)	18 (4.8)	6 (6.3)	
	Once a day	4 (0.9)	4 (1.1)	0 (0.0)	
	Several times during the day	0 (0.0)	0 (0.0)	0 (0.0)	
How often do you eat potatoes (including mashed potatoes)?	Never	30 (6.4)	27 (7.3)	3 (3.2)	0.0001
	1–3 times a month	65 (13.9)	58 (15.6)	7 (7.4)	
	Once a week	111 (23.8)	96 (25.8)	15 (15.8)	
	A few times a week	236 (50.5)	181 (48.7)	55 (57.9)	
	Once a day	21 (4.5)	10 (2.7)	11 (11.6)	
	Several times during the day	4 (0.9)	0 (0.0)	4 (4.2)	
How often do you eat fruit?	Never	0 (0.0)	0 (0.0)	0 (0.0)	0.002
	1–3 times a month	31 (6.6)	21 (5.6)	10 (10.5)	
	Once a week	46 (9.9)	32 (8.6)	14 (14.7)	
	A few times a week	202 (43.3)	165 (44.4)	37 (38.9)	
	Once a day	112 (24.0)	83 (22.3)	29 (30.5)	
	Several times during the day	76 (16.3)	71 (19.1)	5 (5.3)	
How often do you eat vegetables?	Never	0 (0.0)	0 (0.0)	0 (0.0)	0.003
	1–3 times a month	26 (5.6)	16 (4.3)	10 (10.5)	
	Once a week	46 (9.9)	31 (8.3)	15 (15.8)	
	A few times a week	225 (48.2)	177 (47.6)	48 (50.5)	
	Once a day	82 (17.6)	69 (18.5)	13 (13.7)	
	Several times during the day	88 (18.8)	79 (21.2)	9 (9.5)	
How often do you drink fruit, vegetable or fruit and vegetable juices?	Never	0 (0.0)	0 (0.0)	0 (0.0)	ns
	1–3 times a month	104 (22.3)	78 (21)	26 (27.4)	
	Once a week	132 (28.3)	112 (30.1)	20 (21.1)	
	A few times a week	143 (30.6)	114 (30.6)	29 (30.5)	
	Once a day	51 (10.9)	39 (10.5)	12 (12.6)	
	Several times during the day	37 (7.9)	29 (7.8)	8 (8.4)	
How often do you eat sweets, confectionery?	Never	8 (1.7)	8 (2.2)	0(0.0)	0.0001
	1–3 times a month	86 (18.4)	55 (14.8)	31 (32.6)	
	Once a week	71 (15.2)	58 (15.6)	13 (13.7)	
	A few times a week	214 (45.8)	188 (50.5)	26 (27.4)	
	Once a day	59 (12.6)	42 (11.3)	17 (17.9)	
	Several times during the day	29 (6.2)	21 (5.6)	8 (8.4)	
How often do you consume fast food, such as fries, hamburgers, pizza, hot dogs, casseroles?	Never	45 (99.6)	29 (7.8)	16 (16.8)	0.0001
	1–3 times a month	331 (70.9)	287 (77.2)	44 (46.3)	
	Once a week	78 (16.7)	53 (14.2)	25 (26.3)	
	A few times a week	8 (1.7)	3 (0.8)	5 (5.3)	
	Once a day	5 (1.1)	0 (0.0)	5 (5.3)	
	Several times during the day	0 (0.0)	0 (0.0)	0 (0.0)	
How often do you eat fried foods (meat, flour)?	Never	5 (1.1)	2 (0.5)	3 (3.2)	0.0001
	1–3 times a month	50 (10.7)	45 (12.1)	5 (5.3)	
	Once a week	149 (31.9)	124 (33.3)	25 (26.3)	
	A few times a week	233 (49.9)	190 (51.1)	43 (45.3)	
	Once a day	17 (3.6)	8 (2.2)	9 (9.5)	
	Several times during the day	13 (2.8)	3 (0.8)	10 (10.5)	

Differences in behaviour based on the gender of respondents were determined using the χ^2^ test. Statistically significant differences were established at *p* < 0.05.

**Table 5:** The amount of fruit, vegetables and water consumed daily by young adults.

Questions	Levels	Total (%)	Women (%)	Men (%)	*p* Value
How many servings of fruits and vegetables do you eat during the day?	I do not eat them at all	5 (1.1)	0 (0.0)	5 (5.3)	0.0001
	1 serving	143 (30.6)	99 (26.6)	44 (46.3)	
	2 servings	204 (43.7)	169 (45.4)	35 (36.8)	
	3 servings	76 (16.3)	67 (18.0)	9 (9.5)	
	4 servings	24 (5.1)	22 (5.9)	2 (2.1)	
	5 servings and more	15 (3.2)	15 (4.0)	0 (0.0)	
How many glasses of water do you usually drink during the day (mineral, table or other)?	I do not drink water at all	19 (4.1)	13 (3.5)	6 (6.3)	ns
	1 glass	35 (7.5)	28 (7.5)	7 (7.4)	
	2 glasses	81 (17.3)	70 (18.8)	11 (11.6)	
	3 glasses	65 (13.9)	56 (15.1)	9 (9.5)	
	4 glasses	111 (23.8)	91 (24.5)	20 (21.1)	
	5 glasses	61 (13.1)	44 (11.8)	17 (17.9)	
	6 glasses and more	95 (20.3)	70 (18.8)	25 (26.3)	

Differences in behaviour based on the gender of respondents were determined using the χ^2^ test. Statistically significant differences were established at *p* < 0.05.

**Table 6:** Custom sweetening, salting and the preferred method of preparing meat dishes depending on the gender of young adults.

Questions	Levels	Total (%)	Women (%)	Men (%)	*p* Value
Do you sweeten hot drinks, e.g., tea, cocoa, coffee?	No	193 (41.3)	174 (46.8)	19 (20.0)	0.0001
	Yes, I sweeten with one teaspoon of sugar	121 (25.9)	113 (30.4)	8 (8.4)	
	Yes, I sweeten with two or more teaspoons of sugar	153 (32.8)	85 (22.8)	68 (71.6)	
Do you add salt in ready-made dishes at the table?	No	242 (51.8)	202 (54.3)	40 (42.1)	0.002
	Yes, but only sometimes	186 (39.8)	147 (39.5)	39 (41.1)	
	Yes	39 (8.4)	23 (6.2)	16 (16.8)	
How do you usually eat prepared meat dishes? (you can select up to two answers)	Boiled	186 (39.8)	170 (45.7)	16 (16.8)	0.0001
	Stewed	118 (25.3)	86 (23.1)	32 (33.7)	
	Grilled	31 (6.6)	22 (5.9)	9 (9.5)	
	Baked	199 (42.6)	142 (38.2)	57 (60.0)	
	Fried	366 (78.4)	289 (77.7)	77 (81.1)	

Differences in behaviour based on the gender of respondents were determined using the χ^2^ test. Statistically significant differences were established at *p* < 0.05.

## Data Availability

The authors declare that data or models are not deposited in an official repository.
